# Paramedic or GP consultations in primary care: prospective study comparing costs and outcomes

**DOI:** 10.3399/BJGP.2024.0469

**Published:** 2025-02-11

**Authors:** William Hollingworth, Nouf S Gadah-Jeynes, Hazel Taylor, Kirsty Garfield, Sarah Voss, Matthew Booker

**Affiliations:** Department of Population Health Sciences, Bristol Medical School, University of Bristol, Bristol.; Department of Population Health Sciences, Bristol Medical School, University of Bristol, Bristol.; Centre for Trials Research, Cardiff University, Cardiff.; Department of Population Health Sciences, Bristol Medical School, University of Bristol, Bristol.; School of Health and Social Wellbeing, College of Health, Science and Society, University of the West of England, Bristol.; Department of Population Health Sciences, Bristol Medical School, University of Bristol, Bristol.

**Keywords:** cost–benefit analysis, general practitioners, paramedics, patient reported outcome measures, primary health care, workforce

## Abstract

**Background:**

General practice faces pressures because of increased demand and a shortage of GPs. Paramedics in general practice (PGPs) increasingly contribute to managing minor illnesses, conducting home visits, and providing urgent consultations.

**Aim:**

To explore the impact of paramedic consultations on patient-reported experience, safe management, and NHS costs.

**Design and setting:**

Prospective cohort study comparing PGP with GP consultations at 34 GP sites in England.

**Method:**

Eligible participants had a consultation with a PGP (25 PGP sites) or GP (nine non-PGP sites) between May 2022 and February 2023. Questionnaires were provided after the initial consultation and 30 days later. Questionnaires assessed patient experience, outcomes, and perceived safety (PCOQ and PREOS PC), quality of life (EQ-5D-5L), and healthcare use.

**Results:**

Of 715 participants recruited, 489 completed the 30-day questionnaire. No evidence was found that PGP consultations resulted in greater improvement/deterioration in patient-reported health and wellbeing (−0.03, 95% confidence interval [CI] = −0.09 to 0.04); confidence in health provision (−0.05, 95% CI = −0.15 to 0.05); health knowledge (0.05, 95% CI = −0.04 to 0.15); or confidence in the health plan (−0.06, 95% CI = −0.11 to −0.01) over the 30-day period. However, the PGP group reported lower confidence in health provision (mean 4.0 versus 4.5; *P*<0.001), poorer perceptions of practice engagement in safety promotion (median 75 versus 88; *P*<0.001), and more communication problems with staff (17% versus 8%; *P*<0.001) immediately after the initial consultation. Patients receiving PGP consultations reported fewer GP appointments during the 30-day period; however, savings to the NHS were offset by higher use of other healthcare professionals.

**Conclusion:**

Well-designed training and supervision are needed to ensure PGPs have the right knowledge and can clearly convey healthcare plans to patients. While PGPs may reduce GP workload pressure, they do not necessarily reduce NHS costs.

## Introduction

General practice services in England are facing significant pressure owing to increased healthcare demand.^1^^,^^2^ Consultations have been rising by up to 15% annually,^3^ costing the NHS £9 billion,^4^ with a shortage of GPs to meet demand. To address this, there has been a shift towards utilising allied healthcare professionals to support frontline service delivery.^5^

Paramedics have been identified as a professional group that can contribute significantly to general practice, particularly in managing minor illnesses, conducting home visits, and providing urgent consultations.^6^ Primary care initiatives, including the Additional Roles Reimbursement Scheme, recognise that the generalist skillset of paramedics may be well suited to a GP setting.^7^ Recent legislation for paramedic prescribing extends the role of this group. Consequently, there has been a threefold rise in the number of paramedics working in GP services over the past 5 years.^5^ Survey research has noted the wide variation in the education and clinical experience of paramedics working in primary care, and in the clinical work and examinations that they provide.^8^

There is a lack of research on the safety, patient experience, and cost-effectiveness of paramedics working in general practice.^9^ Previous studies have focused on the extended skills needed by paramedics and have made assumptions about their impact on reducing GP workload and costs without empirical evidence.^10^^–^^13^ General practice services are configured around a diverse array of local contexts and challenges, meaning the paramedic skillset is utilised differently across the country.^6^

In the realist evaluation: paramedics in general practice (READY) project,^14^ the same research group evaluated how different models for using paramedics in general practice (PGP) are related to patient and economic outcomes. In this article a component of READY is reported on that prospectively recruited a cohort of participants receiving consultations at practices that did or did not use paramedics. This substudy aimed to compare patient and economic outcomes in the 30 days following PGP or GP consultations. Specifically, the impact on patient-reported outcomes, experience and safe management, and NHS costs/savings was explored.

**Table:** How this fits in

Paramedics are increasingly used in primary care and previous work, although limited, has indicated generally high levels of patient satisfaction. This study found that patients who had a paramedic consultation reported lower confidence in health provision, poorer perceptions of practice engagement in safety promotion, and more communication problems with staff after the initial consultation. Although there was evidence that patients receiving paramedic consultations care had fewer subsequent GP appointments, NHS savings were limited because of higher use of other healthcare professionals. Implications for general practice include improving paramedic training and in situ supervision to ensure paramedics have the right level of medical knowledge and communication skills for work in the primary care setting.

## Method

### Study design and setting

A prospective cohort study was conducted comparing PGP with GP consultations at 34 general practice sites (25 with PGPs and nine without PGPs) in England, based on a published protocol.^14^ A sampling frame was used to ensure representation of sites that varied according to geographical area, practice size, deprivation, rurality, and models of PGP care (see Supplementary Table S1).

### Participant recruitment and data collection

Sites recruited participants between May 2022 and February 2023. Participants were eligible if they were aged ≥16 years, with capacity to give informed consent, registered with a general practice in England, and with understanding of the English language. Practices were asked to approach eligible participants who had a consultation with a paramedic (PGP sites) or GP (non-PGP sites). Consultations could be face to face (surgery or at home) or by telephone/video call. An information sheet, consent form, initial consultation questionnaire, and reply-paid envelope were provided at or soon after the initial consultation. It was planned that participants would be approached by practice administrative staff. However, after feedback from sites, it was agreed that participants could also be approached by clinicians at the time of their appointment. Participants were asked to complete the questionnaire within 24 h of the initial consultation and were sent a follow-up questionnaire by the research team 30 days later. Participants could complete and return the questionnaire via post, online, or by telephone.

### Data

Practice-level data (size, indices of multiple deprivation decile, ethnic group, and urban/rural setting) and local authority-level age-standardised mortality rates were obtained from publicly available data.^15^^,^^16^ The initial consultation questionnaire assessed patient experience and outcomes using the Primary Care Outcomes Questionnaire (PCOQ),^17^ the Patient Reported Experiences and Outcomes of Safety in Primary Care (PREOS-PC compact version),^18^ and the EQ-5D-5L.^19^ The 30-day follow-up questionnaire included the same measures plus the ModRUM questionnaire^20^ to assess use of NHS services.

The PCOQ has four domains: health and wellbeing; confidence in usual health provision; health knowledge and understanding; and confidence in the health plan. Each domain is scored from 1 (severe problems) to 5 (no problems). The PREOS-PC assesses five domains of participant-perceived safety in the past 12 months: practice activation (the degree to which practices are perceived to be engaged in promoting safety); patient activation (the degree to which the patient engages in promoting safety); harm severity; harm burden; and general perceptions of safety. Domains are scored on a 0 (worst) to 100 (best) scale. The PREOS-PC also contains questions about patient experience of specific safety or communication problems in the past 12 months. The EQ-5D-5L comprises five dimensions: mobility; self-care; usual activities; pain/discomfort; and anxiety/depression. EQ-5D-5L responses were mapped^21^ to a scale anchored at 0 (equivalent to death) and 1 (full health), and quality-adjusted life years (QALYs) over the 30-day period were estimated.

Secondary care resource use captured in the ModRUM core questions included the use of accident and emergency, outpatient, inpatient, and day case admissions. It also captured contact with clinicians (for example, GP or allied health professionals), modality (for example, face to face, virtual, or home visits), and in-depth questions on prescribed medications. Resources were valued in 2021–2022 UK prices.

### Study size

The study aimed to recruit 1104 participants across 24 practices. Given assumptions (50% follow-up; 0.02 intracluster correlation coefficient; 0.65 cluster size coefficient of variation; two-sided test; 0.05 significance level), the study was powered to detect differences between two different models of PGP care (see Supplementary Table S1) or GP care. This would require 138 participants in each model of care (totalling 552 with three PGP models and a GP model with complete data and ≥90% power) to identify a 0.5 standard deviation (SD) difference between two groups on PCOQ change scores. However, owing to slow recruitment, 34 sites recruited 715 eligible participants, of whom 489 (89% of the intended sample size) completed the 30-day questionnaire.

### Analytical methods

In this article, the focus is on the PGP versus GP comparison rather than comparisons between different models of PGP care. Patient data were collected using REDCAP software and statistical analyses were conducted using Stata/IC (version ≥14.0).

Baseline characteristics include the practice-level characteristics described above and the participant-level characteristics: age, sex, ethnic group, and appointment mode. Multilevel models were fitted to adjust for potential bias because of differences in practice and participant characteristics. GP practice was fitted as a random effect and all other covariates fitted as fixed effects. Multilevel models included the participant-level variables: initial consultation questionnaire scores, age, sex, ethnic group (White or other ethnic group), and all practice and local authority-level variables. As a result of the high skewness of most PREOS-PC scores, a multilevel model was only fitted for the practice activation domain. A multilevel logistic regression model was also fitted for the PREOS-PC general perceptions of safety visual analogue scale (VAS) score dichotomised at <90 versus ≥90. Missing data were imputed according to user guides for the PCOQ and PREOS-PC.^18^^,^^22^

The primary economic analysis estimated the incremental NHS costs of 30-day episodes of care following PGP and GP consultations. In secondary analyses a cost–utility analysis was conducted estimating the incremental net monetary benefit (iNMB) from the NHS perspective at the threshold of £20 000 per QALY gained.^23^

## Results

### Participants and practices

**Figure 1.**
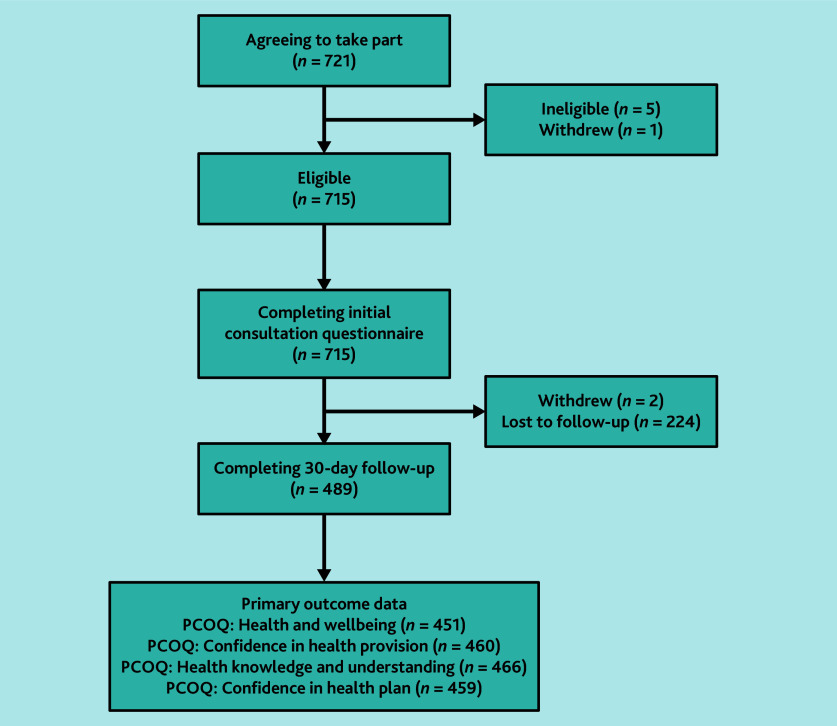
Patients approached and participating in the study. PCOQ = Primary Care Outcomes Questionnaire.

There were 715 participants who were eligible and completed the initial consultation questionnaire (Figure 1). PGP sites were substantially larger than non-PGP sites (median size 14 671 versus 7900 registered patients) and had a lower mean percentage of registered participants recorded as other ethnic group (median 3.9% versus 11.2%) (Table 1).

**Table 1.** Site, participant, and appointment characteristics

**Characteristic**	**PGP sites, *N* = 25**	**Non-PGP sites, *N* = 9**
**Site characteristic**		
Practice size, median (range)	14 671 (3965–44 964)	7900 (4710–31 860)
IMD decile, median (range)	7 (1–10)	8 (5–10)
ASMR, median (range)	1057 (761–1315)	1030 (802–1065)
Other ethnic group, %, median (range)	3.9 (1.1–27.5)	11.2 (1.4–49.1)
Urban sites	21 (84)	7 (78)

**Participant characteristic**	***N* = 487^a^**	***N* = 228^a^**
Participants with 30 days’ follow-up	341 (70)	148 (65)
Age, median (range)	58 (44–71)	61 (47–72)
Sex, male	143 (29)	59 (26)
Ethnic group		
White	450 (96)	206 (92)
Other	20 (4)	18 (8)

**Appointment characteristic^b^**	***N* = 481**	***N* = 227**
Mode of appointment		
Face to face at home	27 (6)	7 (3)
Face to face at surgery	351 (73)	173 (76)
Telephone/video/text/email	103 (21)	45 (20)
Appointment with^b^		
Paramedic	412 (86)	4 (2)
GP	36 (7)	208 (92)
Other	33 (7)	15 (7)

*Values are n (%) unless stated otherwise.*

a *Owing to missing data, participant characteristics were available for 695 (for ethnic group), 703 (for age), and 706 (for sex) responders.*

b *Appointment characteristics were self-reported by participants. Not every participant reported the mode of appointment and some apparently misidentified the profession of the healthcare professional. ASMR = age-standardised mortality rate. IMD = indices of multiple deprivation. PGP = paramedics in general practice.*

Participants at PGP sites were slightly younger (median age 58 versus 61 years) with similar sex and ethnic group distributions. The majority of appointments took place face to face at the surgery (73% [*n* = 351/481] at PGP sites versus 76% [*n* = 173/227] at non-PGP sites). Although all participants at PGP sites were seen initially by PGPs, a minority reported having seen a GP (*n* = 36/481; 7%) or other healthcare professional (*n* = 33/481; 7%) at their initial consultation, presumably due to inaccurate patient recall. In total, 489 of 715 (68%) participants completed the 30-day questionnaire; response rates were higher at PGP practices (*n* = 341/487; 70%) than non-PGP practices (*n* = 148/228; 65%). The median age of responders to the 30-day questionnaires was marginally older than that of all participants recruited (see Supplementary Table S2).

### Initial consultation

PCOQ scores were generally high (median ≥4) after the initial consultation with PGPs and GPs in all four domains (Table 2). However, a difference was observed in the ‘confidence in health provision’ domain, with lower scores (that is, less confidence in the doctors and nurses you usually see) observed in the PGP group (median 4.0 versus 4.5; *P*<0.001). On three domains (harm severity, harm burden, and general perceptions of safety), median PREOS-PC scores were very high (median 100) after the initial consultation, with both groups reporting very few concerns about care received at the GP surgery in the past 12 months.

**Table 2.** Participant-reported outcomes after the initial consultation: PGP versus GP

**Outcome measure**	**PGP**		**GP**		***P*-value^b^**

	***n***	**Median (IQR) or mean (SE)^a^**	***n***	**Median (IQR) or mean (SE)^a^**	
**PCOQ**					
Health and wellbeing	460	4.0 (3.3–4.5)	218	4.1 (3.5–4.5)	0.030
Confidence in provision	461	4.0 (3.5–4.8)	221	4.5 (4.0–5.0)	<0.001
Knowledge and understanding	463	4.8 (4.0–5.0)	221	4.8 (4.0–5.0)	0.779
Confidence in health plan	457	4.3 (3.8–4.8)	220	4.3 (4.0–4.8)	0.627

**PREOS-PC**					
Practice activation	412	75 (56–94)	204	88 (75–100)	<0.001
Patient activation	282	25 (0–63)	131	38 (0–63)	0.545
Patient harm severity	411	100 (100–100)	198	100 (100–100)	0.027
Patient harm burden	409	100 (100–100)	197	100 (100–100)	0.045
General perceptions of safety	400	100 (90–100)	201	100 (90–100)	0.002

**EQ-5D-5L**					
Utility score	457	0.715 (0.243)	225	0.770 (0.124)	0.003
VAS score	477	67.4 (0.966)	224	70.7 (1.301)	0.052

a *Median (IQR) for PCOQ and PREOS-PC, mean (SE) for EQ-5D-5L.*

b *Using Mann-Whitney U tests for comparison of medians, T-Test for comparison of means. IQR = interquartile range PCOQ = Primary Care Outcomes Questionnaire. PGP = paramedics in general practice. PREOS-PC = Patient Reported Experiences and Outcomes of Safety in Primary Care. SE = standard error. VAS = visual analogue scale.*

PREOS-PC practice activation scores after the initial consultation were lower, particularly in the PGP group (median 75 PGP versus 88 GP group; *P*<0.001) (Table 2). Patient activation scores were low in both groups (median 25 PGP versus 38 GP group; *P* = 0.545). EQ-5D-5L quality of life scores were lower in the PGP group (mean 0.715 PGP versus 0.770 GP; *P* = 0.003).

Reports of specific safety problems in the past 12 months were relatively rare and similar across PGP and GP groups (Table 3). Problems with medications prescribed in the past 12 months (8% PGP [*n* = 38/487] and 6% GP group [*n* = 14/228]) were most commonly reported.

**Table 3.** Safety and communication problems in the past 12 months reported after initial consultation

**Problem**	**PGP**	**GP**	***P*-value^a^**
**Safety problem in the last 12 months,*n* (%)**	***N* = 487**	***N* = 228**	
Diagnosis	34 (7)	12 (5)	0.419
Medication prescribed	38 (8)	14 (6)	0.537
Other treatments prescribed	14 (3)	5 (2)	0.804
Vaccines prescribed	12 (2)	4 (2)	0.791
Blood and lab tests	21 (4)	8 (4)	0.687
Diagnosis and follow-up tests	18 (4)	6 (3)	0.514
Appointments	34 (7)	7 (3)	0.031
Health records	22 (5)	4 (2)	0.085

**Communication problem in the last 12 months, *n* (%)**			
Between you and healthcare staff	71/412 (17)	16/203 (8)	<0.001
Among healthcare staff in the GP surgery	52/409 (13)	14/202 (7)	0.037
Between GP staff and other professionals	55/408 (13)	20/200 (10)	>0.99

a *Using Fisher’s exact test for categorical outcomes.*

Communication problems were reported more frequently, for example, 17% of participants in the PGP group and 8% of participants in the GP group reported communication problems between patients and staff in the past 12 months (*P*<0.001) (Table 3). A slightly higher percentage of patients in the PGP group reported not being able to get an appointment when they needed one (7% PGP [*n* = 34/487] and 3% GP [*n* = 7/228]; *P* = 0.031).

### Thirty days and change between initial consultation and 30 days

Responses to the 30-day questionnaire followed a similar pattern to the initial consultation responses (see Supplementary Table S3). The PCOQ health and wellbeing domain change scores indicated small improvements in both groups. Participant perceptions of practice activation remained lower in the PGP group. Results from the multilevel analyses (Table 4) revealed generally small and statistically insignificant differences between the PGP and GP groups in PCOQ change scores from initial consultation to 30 days later. Confidence in the health plan deteriorated slightly more in the PGP group (difference in change score −0.06, 95% confidence interval [CI] = −0.11 to −0.01; *P* = 0.012). There was a statistically significant difference in the PREOS-PC practice activation scores at day 30, which were lower in the PGP group (adjusted difference in means −4.5, 95% CI = −7.1 to −2.0), suggesting that the PGP group increasingly felt that their practices were less engaged in promoting safety.

**Table 4.** Adjusted difference in mean (95% CI) PCOQ change scores and PREOS-PC scores between PGP and GP groups^a^

**Outcome measure**	**PGP versus GP**	***P*-value**
**Change in PCOQ (30 day, initial score), mean difference in change scores (95% CI)**		
Health and wellbeing, *n* = 433	−0.03 (−0.09 to 0.04)	0.465
Confidence in health provision, *n* = 441	−0.05 (−0.15 to 0.05)	0.302
Health knowledge and understanding, *n* = 447	0.05 (−0.04 to 0.15)	0.286
Confidence in health plan, *n* = 440	−0.06 (−0.11 to −0.01)	0.012
**PREOS-PC at day 30**		
Practice activation, difference in means (95% CI), *n* = 389	−4.53 (−7.07 to −2.00)	<0.001
General perceptions of safety, odds ratio^b^(95% CI), *n* = 386	1.34 (0.73 to 2.47)	0.348

a *Multilevel modelling adjusting for the participant-level factors: initial score, age (continuous), sex, ethnic group (White or other ethnic group), and for the practice-level factors: age standardised mortality rate (continuous), % other ethnic group (continuous), urban versus rural, practice size (small, medium, or large) and deprivation decile (1–3, 4–7, or 8–10), with site fitted as a random effect.*

b *Adjusted odds ratio for a VAS <90 versus ≥90 obtained from a multilevel logistic regression model. PCOQ = Primary Care Outcomes Questionnaire. PGP = paramedics in general practice. PREOS-PC = Patient Reported Experiences and Outcomes of Safety in Primary Care. VAS = visual analogue scale.*

### Economic analysis

Mean per participant NHS costs over the 30-day episode of care were marginally higher in the PGP group: £345.41 versus £315.55 (Table 5). Mean reported GP consultations were lower in the PGP group (1.25 versus 1.73), but this saving was largely offset by higher use of other healthcare professionals (including PGPs). In multilevel regression (see Supplementary Table S4), PGP consultations were not associated with a statistically significant difference in overall NHS costs. Overall adjusted mean NHS costs were £11.89 more following PGP consultations (95% CI = −£160.90 to £184.10). The GP group reported marginally higher utility scores at the initial consultation and 30-day follow-up time points (Table 2 and Supplementary Table S3). However, there was no difference in incremental adjusted QALYs between the groups (0.000, 95% CI = −0.001 to 0.002; Supplementary Table S4). At a willingness to pay threshold of £20 000 per QALY gained, the iNMB of a PGP consultation was −£11.61 (95% CI = −186.34 to 163.13), suggesting that PGP consultations were not more cost-effective than GP consultations.

**Table 5.** Participant-reported healthcare use during the 30-day episode after the initial consultation

**Resource use**	**PGP, *N* = 287^a^**			**GP, *N* = 124^a^**		

	**Mean resource use**	**Mean cost, £**	**SD, cost**	**Mean resource use**	**Mean cost, £**	**SD, cost**
**Primary healthcare resource use**						
GP	1.25	22.30	27.63	1.73	29.76	24.22
Other HCP^b^	1.10	12.03	17.56	0.67	4.15	6.02
Prescriptions	2.61	22.30	42.66	2.57	23.54	42.21

**Secondary healthcare resource use**						
Outpatient	0.73	130.94	217.75	0.69	125.82	206.69
A&E	0.13	38.78	152.56	0.09	27.05	95.33
Admissions^c^	0.19	119.07	662.61	0.16	105.24	742.79

**Total NHS costs**		345.41	806.28		315.55	838.43

a *Patients with complete NHS resource-use data.*

b *HCP: includes nurses, paramedics, and other non-GP contacts.*

c *Includes day case patients and overnight stays. A&E = accident and emergency. HCP = healthcare professional. PGP = paramedics in general practice. SD = standard deviation.*

## Discussion

### Summary

When compared with GP consultations, no evidence was found in this study that PGP consultations resulted in improvements or deterioration in patient-reported health and wellbeing, health knowledge, and understanding, or confidence in health provision over the 30 days after an initial consultation. However, the PGP group reported lower confidence in health provision, poorer perceptions of practice engagement in safety promotion, and more communication problems between patients and staff immediately after the initial consultation. Further work is required to understand whether this reflects care at the practice in general or specifically the care provided during the initial consultation. Although there was some evidence that patients receiving PGP consultations care had fewer GP appointments, savings to the NHS were offset by higher use of other healthcare professionals. Therefore, although PGP consultations may have reduced GP workload pressure they did not reduce NHS costs.

### Strengths and limitations

This large prospective cohort study has described the association between PGP consultations and the costs, quality, and safety of care. The authors were able to explore how participant perceptions of care and health-related quality of life changed over time. However, the study did not achieve the sample size target. Conducting the study during the recovery from the COVID-19 pandemic had an impact on recruitment. The authors initially aimed to explore the association between different models of PGP care and participant-reported outcomes; however, small numbers of participants in some model configurations precluded meaningful comparisons. The study recruited English-language speakers and therefore findings cannot be extrapolated more broadly.

The appropriate choice of comparator group is challenging as PGPs fulfilled different roles in different practices. In this study, GP consultations were selected as PGPs were often employed to deliver care typically provided by GPs. Despite adjusting for participant and practice characteristics, the observational nature of the study makes it difficult to be certain whether the differences observed in outcomes were attributable to the initial appointment (with a PGP or GP) or other unobserved factors. Data on the specific conditions seen by PGPs or GPs were not available. However, it is of note that EQ-5D-5L scores were lower among patients in the PGP group at the initial appointment, indicating that they had, on average, worse health-related quality of life. The PCOQ asks questions about primary care outcomes ‘at the moment’ whereas the PREOS-PC frames questions about safety ‘in the last 12 months’. Therefore, the PCOQ might be considered more likely to identify immediate concerns with the initial consultation, whereas the PREOS-PC might reflect longstanding views about the safety of care at the practice.

The estimation of NHS resource use relied on participant recall. Furthermore, approximately one-third of participants did not respond to the 30-day questionnaire, potentially introducing response bias. PGP and GP booking slot duration typically did not differ within practices; however, consultation times were not routinely recorded. Therefore, the authors did not accurately know whether PGPs spent longer with patients. The data in the study shed light on the redistribution of patient-facing work between GPs and PGPs; however, they do not reflect other work (for example, provision of training and supervision) that will undoubtedly affect the value of PGPs in relieving GP workload pressures. Although individual patient data were not available on the time between requesting and receiving an appointment, it is of note that patients in the PGP group were more likely to report problems in getting an appointment when needed.

### Comparison with existing literature

A systematic review^9^ identified a small number of studies that evaluated patient satisfaction with paramedic care in primary care home visits. The review concluded that, although there were high satisfaction levels with paramedic care, a minority of participants remained keen to be assessed by their GP and/or remained unclear about the purpose of the paramedic assessment. The current study was broader, including PGP consultations at the surgery and telemedicine, and the current findings that participants who had seen a PGP had lower confidence in health provision after the consultation add to this evidence base. Although this finding raises concerns, the authors cannot directly attribute it to paramedic consultations because the PCOQ questions typically refer to ‘doctors and nurses you usually see’.

Previous work by the same research group has highlighted the need for more evidence on the effect of paramedics on participant safety.^6^ The current findings on participant safety are novel and indicate that participants who received care from PGPs had more concerns about practice activation. The practice activation domain includes questions about availability of practitioners to talk to and provision of information about the side effects of treatment. Again, these concerns may relate more generally to the practice rather than specifically to the paramedic but are worth further investigation.

It is clear from previous work^8^^,^^24^ that the successful introduction of paramedics into primary care will be dependent on PGP training and clinical experience; organisational factors, such as the provision of clinical supervision and the integration of PGPs within the practice; and the complexity of patients and clinical activities that PGPs are assigned to. In this article, the overall patient experience, outcomes, and NHS costs across a broad spectrum of PGP care models are described.

### Implications for research and practice

Additional research to see whether the current findings are replicated in other primary care settings is important. Such research might use bespoke questions about the quality and safety of care at the most recent consultation. This would help tease apart practice-related and paramedic-related concerns. Larger studies with longer follow-up are needed to more fully evaluate rare outcomes (for example, hospital admissions) that may ultimately define the safety and cost-effectiveness of PGPs. Further research could also describe in more detail the impact of PGPs on appointment accessibility, consultation times, and requirements for GPs to train and supervise new PGP staff. Future work involving larger numbers of GP practices that employ PGPs with different degrees of integration (for example, rotational versus full time) and for different clinical caseloads (for example, from minor illness and routine home visits through to largely autonomous prescribers with few patient restrictions) is needed to better define the appropriate roles of PGPs in general practice.

If the current findings are replicated, there are important implications for general practice. These include careful planning in how paramedics are deployed in primary care so that they can quickly gain the trust of the patients that they see. They also include well-designed paramedic training and in situ supervision to ensure that they have the right medical knowledge and can clearly convey healthcare plans to patients. There may also be a place for better communication between the practice and patients about the role of paramedics within their practice to manage expectations and provide reassurance.

## Data

The participants of this study did not give written consent for their data to be shared publicly, so owing to the sensitive nature of the research supporting data are not available.
